# Bioactivity of Natural and Engineered Antimicrobial Peptides from Venom of the Scorpions *Urodacus yaschenkoi* and *U. manicatus*

**DOI:** 10.3390/toxins9010022

**Published:** 2017-01-06

**Authors:** Karen Luna-Ramirez, Miray Tonk, Mohammad Rahnamaeian, Andreas Vilcinskas

**Affiliations:** 1 Fraunhofer Institute for Molecular Biology and Applied Ecology, Winchester Strasse 2, D-35394 Giessen, Germany; karenlunarmrz@gmail.com (K.L.-R.); miray.tonk@ime.fraunhofer.de (M.T.); mohammad.rahnamaeian@ime.fraunhofer.de (M.R.); 2 Institute for Insect Biotechnology, Justus Liebig University of Giessen, D-35392 Giessen, Germany

**Keywords:** antimicrobial peptides, venom, hemolysis, therapeutic index, scorpions

## Abstract

The spread of multidrug-resistant human pathogens has drawn attention towards antimicrobial peptides (AMPs), which are major players in the innate immune systems of many organisms, including vertebrates, invertebrates, plants and microbes. Scorpion venom is an abundant source of novel and potent AMPs. Here, we investigated natural and engineered AMPs from the scorpions *Urodacus yaschenkoi* and *U. manicatus* to determine their antimicrobial spectra as well as their hemolytic/cytotoxic activity. None of the AMPs were active against fungi, but many of them were active at low concentrations (0.25–30 µM) against seven different bacteria. Hemolytic and cytotoxic activities were determined using pig erythrocytes and baby hamster kidney cells, respectively. The amino acid substitutions in the engineered AMPs did not inhibit cytotoxicity, but reduced hemolysis and therefore increased the therapeutic indices. The phylogenetic analysis of scorpion AMPs revealed they are closely related and the GXK motif is highly conserved. The engineered scorpion AMPs offer a promising alternative for the treatment of multidrug-resistant bacterial infections and could be modified further to reduce their hemolytic/cytotoxic activity.

## 1. Introduction 

The emergence and spread of bacteria that are resistant to many routinely-used antibiotics has driven research into the development of new classes of antimicrobial agents with high antibacterial efficacy and new modes of action. Naturally occurring antimicrobial peptides (AMPs) are essential components of the innate immune system in many different organisms, and they are characterized by diverse targets and mechanisms of action, broad-spectrum activity and only rare instances of resistance [1,2,3,4]. AMP gene families have expanded and diversified particularly among the arthropods, compensating for the lack of an adaptive immune system [5].

AMPs have been isolated and characterized from many arthropods, including insects [6,7], ticks [8,9] and scorpions [10,11,12]. Natural AMPs generally have short, cationic, amphipathic and α-helical structures that interact preferentially with microbial membranes via electrostatic contacts [13,14]. Increasing the net positive charge and hydrophobicity enhances the antibacterial activity of AMPs due to the greater affinity for the negatively-charged bacterial membrane [15]. However, the therapeutic value of AMPs is limited by their hemolytic activity, which is particularly prevalent in the case of scorpion AMPs. The therapeutic index of AMPs can be improved by reducing their hemolytic activity, which can be achieved by engineering new versions with suitable amino acid substitutions [16]. 

Here, we describe the functional characterization of natural and engineered versions of the scorpion AMPs UyCT1, UyCT3, UyCT5, Uy17, Uy192 and Uy234 from *Urodacus yaschenkoi*, and Um2, Um3, Um4 and Um5 from *U. manicatus* [17,18,19,20]. The AMPs were tested for antifungal, antibacterial, hemolytic and cytotoxic activities in order to identify more potent and selective versions with a higher therapeutic index.

## 2. Results

### 2.1. Peptide Engineering

To engineer enhanced scorpion AMPs, we aimed to keep the conserved residues and retain the amphipathic properties of the engineered sequences (Table 1). 

**Figure 1:**
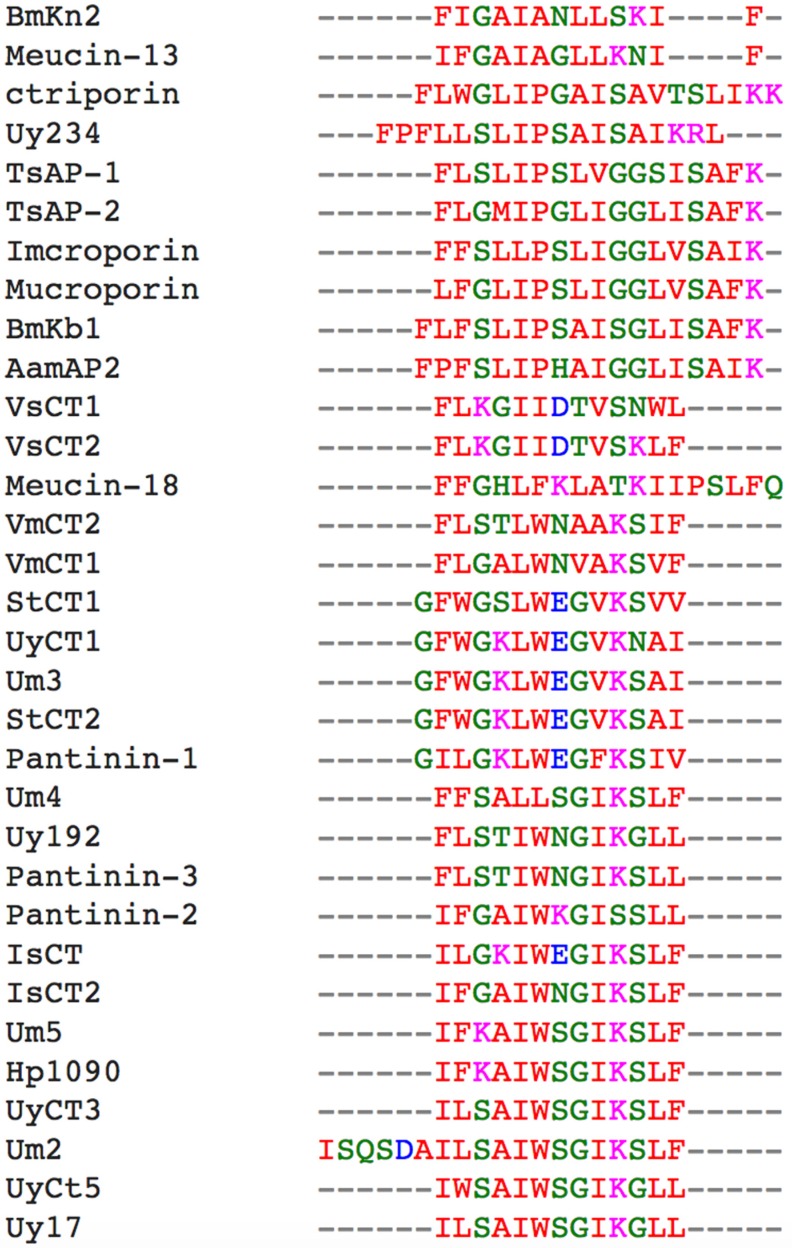
Amino acid sequence alignment of the scorpion AMPs by Clustal Omega. Accession numbers: *Scorpiops tibetanus*: StCT1 (PODJO3), StCT2 (PDJO4); *Pandinus imperator*: Pantinin 1 (AGK88380), Pantinin 2 (AGK88381), Pantinin 3 (AGK88382); *Heterometrus petersii*: Hp1090 (P0DJ02); *Opisthacanthus madagascariensis*: IsCT1 (Q8MMJ7), IsCT2 (Q8MTX2); *Vaejovis mexicanus*: VmCT1 precursor (AFH87944), VmCT2 precursor (AFH87945); *Mesobuthus eupeus*: Meucin-18 (E4VP50); *Chaerilus tricostatus*: Ctriporin (G1FE62); *Mesobuthus martensii*: BmKb1 (Q718F4), BmKn2 (Q6JQN2); *Androctonus amoreuxi*: AamAP2 (G8YYA6); *Lychas mucronatus*: Mucroporin (B9UIY3); *Isometrus maculatus*: Imcroporin (C7B247); *Tityus serrulatus*: TsAP-1 precursor (CCQ98791), TsAP-2 precursor (CCQ98792); *Mesobuthus eupeus*: Meucin-13 (E4VP07); *Vaejovis subcristatus*: VsCT1 and VsCT2 (EST database: dbEST JZ818318-JZ818449); *Urodacus yaschenkoi*: UyCT1 (AGA82754), UyCT3 (AGA82755), UyCT5 (AGA82756); *Urodacus manicatus*: Um2 (JAA98072), Um3 (JAA98071), Um4 (JAA98070), Um5 (JAA98069).

The aligned UyCT peptides revealed a conserved Trp residue in the middle of the sequence and the G(V/I)K motif near the C-terminus, and these elements were retained (Figure 1). 

The mutations were made by interchanging other amino acids and inserting positively charged residues to increase the net positive charge and hence bacterial membrane affinity (Table 2).

### 2.2. Antimicrobial Assays

The antimicrobial activities of 16 scorpion-derived natural and engineered AMPs were evaluated against the Gram-positive bacteria *L. monocytogenes*, *L. grayi*, *L. fleischmannii*, *S. aureus* and *M. luteus*, and the Gram-negative bacteria *E. coli* and *P. aeruginosa*. *M. luteus* was the most susceptible species, and it was completely inhibited in the presence of 0.25 μM D10 or D11 (Table 3). Most of the AMPs showed no effect against *P. aeruginosa* even at the highest concentration we tested (250 µM), but UyCT1 and D11 were able to inhibit the growth of this species at 60 μM and 30 μM, respectively. UyCT5, Uy192, Um3 and Um4 were the most broadly-active AMPs, inhibiting all the bacterial species we tested except *P. aeruginosa*. D10, D11 and Um5 displayed the lowest MIC values. Table 3 summarizes the MIC values, cytotoxicity (ECx) and hemolytic activity (HCx) of the most active AMPs.

Antifungal activity was assessed using two major phytopathogenic fungi, *F. culmorum* and *F. graminearum*. None of the tested scorpion peptides showed any effect on fungi, even at the concentration of 200 µM. 

### 2.3. Hemolytic Assays

The hemolytic effect of the scorpion AMPs was tested by exposing pig erythrocytes to 100 µM of each peptide (Table 3). The percentage of hemolysis was calculated using 10% Triton X-100 as a positive control (100% hemolysis). Um4 and D4 were the least hemolytic peptides, causing 9% and 1.7% hemolysis at the highest tested concentration (100 μM), respectively, but it was not technically possible to calculate HC_50_ values because the data did not fit the curve. Engineered peptides D5, D10 and D11 caused limited hemolysis (~10% each). The most hemolytic peptides were UyCT5 and D2, which caused 70% and 64% hemolysis, respectively. In contrast, Um4 and D4 showed less than 1% hemolytic activity. The HC_50_ values for the natural and engineered AMPs ranged from 20 to 142 µM and 24 to 1700 µM, respectively. 

### 2.4. Cytotoxicity Assays

The natural and engineered AMPs were mildly cytotoxic against BHK-21 cells (Table 3). Engineered peptides D2, D4, D5 and D10 were less cytotoxic than the other AMPs. D4 and D10 are engineered versions of UyCT1. The engineered peptides showed more desirable EC_50_ and HC_50_ values than the natural peptide, indicating that the rationale for the engineering strategy was correct, producing safer and more potent molecules. The least cytotoxic natural AMPs were Uy17, Uy192, Um3 and Um4. 

### 2.5. Phylogenetic Analysis

**Figure 2:**
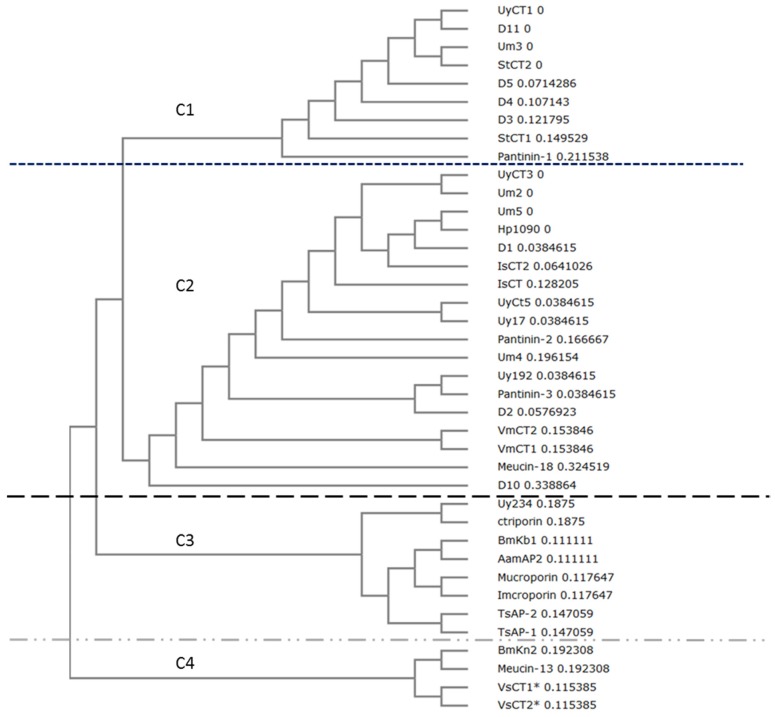
Phylogenetic analysis of natural and designed scorpion AMPs with ClustalW2 Phylogeny using the Nexus UPGMA model. Tree shows the cladistic relationship between AMPs. Branch length is included beside the name of the peptide. Accession numbers: *Scorpiops tibetanus*: StCT1 (PODJO3), StCT2 (PDJO4); *Pandinus imperator*: Pantinin 1 (AGK88380), Pantinin 2 (AGK88381), Pantinin 3 (AGK88382); *Heterometrus petersii*: Hp1090 (P0DJ02); *Opisthacanthus madagascariensis*: IsCT1 (Q8MMJ7), IsCT2 (Q8MTX2); *Vaejovis mexicanus*: VmCT1 precursor (AFH87944), VmCT2 precursor (AFH87945); *Mesobuthus eupeus*: Meucin-18 (E4VP50); *Chaerilus tricostatus*: Ctriporin (G1FE62); *Mesobuthus martensii*: BmKb1 (Q718F4), BmKn2 (Q6JQN2); *Androctonus amoreuxi*: AamAP2 (G8YYA6); *Lychas mucronatus*: Mucroporin (B9UIY3); *Isometrus maculatus*: Imcroporin (C7B247); *Tityus serrulatus*: TsAP-1 precursor (CCQ98791), TsAP-2 precursor (CCQ98792); *Mesobuthus eupeus*: Meucin-13 (E4VP07); *Vaejovis subcristatus*: VsCT1 and VsCT2 (EST database: dbEST JZ818318-JZ818449); *Urodacus yaschenkoi*: UyCT1 (AGA82754), UyCT3 (AGA82755), UyCT5 (AGA82756); *Urodacus manicatus*: Um2 (JAA98072), Um3 (JAA98071), Um4 (JAA98070), Um5 (JAA98069). Identity of the clade is represented by C#, in example C1 = clade 1.

A phylogenetic tree comprising all of the reported active natural scorpion AMPs and the tested herein was built using ClustalW2 Phylogeny based on the Nexus UPGMA model, revealing four major clades (Figure 2). The first clade comprised sequences similar to UyCT1, including two AMPs from *Scorpiops tibetanus* (StCT1 and STCT2) and Um3, which differs by only one amino acid from UyCT1. Pantinin-1 form *Pandinus cavimanus* is also included in this clade. The second clade contained sequences similar to UyCT3. This was more diverse, containing sequences from *U. yaschenkoi* (UyCT3, UyCT5, Uy17, Uy192), *U. manicatus* (Um2, Um5, Um4), *Pandinus cavimanus* (Pantinin-2, Pantinin-3)*, Heterometrus petersii* (Hp1090)*, Opisthacanthus madagascariensis* (IsCT1, IsCT2)*, Mesobuthus eupeus* (Meucin-18) and *Vaejovis mexicanus* (VmCT1, VmCT2). Third clade comprised the longer AMPs (17–19 residues, whereas all the other AMPs were 13–14 residues in length). All the AMPs in this clade are secreted from scorpions representing the Buthidae family, except ctriporin which represents the Chaerilidae family and Uy234 from the Urodacidae family. The fourth clade contained AMPs 13 residues in length, including VsCT1 and VsCT2 from *Vaejovis subcristatus*, which showed no antibacterial activity [21].

## 3. Discussion

Scorpion venoms contain hundreds of different compounds that target membranes, ion channels and receptors [22,23]. One class of compounds, known as “non-disulfide-bridged peptides”, comprises short peptides with diverse properties including antimicrobial activity, lytic activity and the potentiation of bradykinin and cell signaling [24]. These peptides are considered to belong to the broader category of antimicrobial peptides (AMPs), an important component of innate immunity in many different organisms, due to their ability to inhibit the growth of pathogens including multidrug-resistant bacteria at MIC values as low as 1 µM [18,19,25]. Scorpion venom AMPs therefore provide a rich source of potential new antimicrobial compounds that could be used to address the increasing prevalence of pathogens resistant to conventional antibiotics. However, many scorpion AMPs also cause undesirable levels of hemolysis and cytotoxicity, so the use of natural peptides would cause unacceptable side effects. It may nevertheless be possible to exploit these peptides if peptide engineering can be used to balance potency with increased selectivity against pathogens [26]. 

Natural AMPs in scorpion venom are highly conserved (Figure 1) as shown by their phylogenetic clustering (Figure 2), and in some cases the difference between AMPs can be as little as one or two residues. Interestingly, such small differences in sequence often result in much larger differences in bioactivity. For example, UyCT1 from *U. yaschenkoi* [18] and Um3 from *U. manicatus* [20] differ at only one residue (S12N), but this substitution makes UyCT1 more potent that Um3 (Table 3). The degree of conservation among scorpion venom AMPs is such that some diverse species produce identical AMPs, e.g., StCT2 from *S. tibetanus* [27] is identical to Um3 from *U. manicatus*, and UyCT3 from the Australian scorpion *U. yaschenkoi* [18] is identical to OcyC1 from the Brazilian scorpion *Opisthacanthus cayaporum* [28]. UyCT3 differs from Um5 at two positions (L2F and S3K), and the nature of the substitutions increases the net positive charge on Um5, increasing its activity against all the bacteria we tested (Table 3). These natural substitutions led to our hypothesis that a rational engineering approach could be used to modify scorpion AMPs to increase their potency and specificity for microbial membranes, thereby reducing their hemolytic activity and cytotoxicity towards mammalian cells and thus improving their therapeutic index. 

Here, we compared the bioactivity of natural and engineered scorpion AMPs to investigate their suitability as therapeutic leads. In order to preserve the secondary structure of the natural AMPs as far as possible, changes were only made in non-essential residues and their purpose was to increase the positive net charge (Table 2). The UyCT peptides showed low MIC values (1–15 µM) against several human pathogens, including multidrug resistant bacteria, but they also caused mild hemolysis [19]. Rational engineering was most successful in the case of UyCT1, where the addition of a C-terminal oligo-lysine tail to generate engineered derivative D11 reduced the hemolytic activity to negligible levels (HC_50_ = 1110 µM) compared to the parental UyCT1 (HC_50_ = 142.5 µM). The increase in net positive charge and the bulky side chains made the peptide more potent and also more selective towards bacteria (Table 3). 

Another potential way to improve selective antimicrobial activity is to modulate the charge distribution of amphipathic helices, i.e., increase the hydrophobicity of the hydrophobic face and/or the hydrophilicity of the hydrophilic face [29]. We increased the net positive charge of UyCT1 by adding lysine residues either at the C-terminus or distributed along the parent sequence (Table 2). As stated above, the engineered peptide D11 (C-terminal oligo-lysine tail) had a much lower MIC than the parental peptide UyCT1 (~1 µM compared to ~4 µM) against all seven bacteria tested (Table 3), probably reflecting the formation of a more effective membrane-transducing domain [30]. In contrast, D12 (based on UyCT1 but with distributed lysine residues) was totally inactive (data not shown). Similar results were observed with UyCT1 when we replaced the negatively charged glutamic acid residue with lysine (E8K) to generate the D4 derivative. The D4 peptide was less potent than UyCT1 (Table 3) but also showed no hemolytic activity (Table 3). Similarly, D5 was derived from UyCT1 by replacing tryptophan with leucine (W6L) and it showed only ~25% of the activity of the parental AMP (Table 3), probably because the presence of the large tryptophan side chain enhances the membrane purterbation [31]. These findings suggest that an increase in the net positive charge can increase the potency of AMPs but only if the charge is concentrated at the end of the peptide and not distributed throughout the sequence. 

The engineered peptides D5, D10 and D11 showed a significant loss of hemolytic activity compared to their parent sequences (Table 3), substantially increasing the corresponding therapeutic index, especially in the case of D11 (Table 4). D5 is a derivative of UyCT1 with W7L and N12K substitutions, and D10 is a derivative of Uy234 with the addition of four scattered lysine residues (Table 2). The hemolytic activity of these engineered peptides was 100-fold lower than the parent peptides (Table 3). However, more sophisticated engineering approaches are required to reduce HC_50_ values. For example, the naturally occurring peptides UyCT3 and Um5 differ at two residues (positions 2 and 3) but their HC_50_ values are almost identical (~60 µM), whereas UyCT1 and Um3 differ only at position 12 and the HC_50_ values are 142.5 and 126.2 µM, respectively. The hemolytic effect of AMPs may depend more on the stereochemistry of the residues than the nature of the substitution [16], so L-to-D replacements may offer a better strategy to reduce the hemolytic activity of scorpion AMPs [32].

UyCT3 and D1 differed at two positions (L2F and S3G) but behaved similarly in terms of antibacterial and hemolytic activities (Table 3), possibly because both leucine and phenylalanine are lipophilic with neutral nonpolar side-chains. Similarly, replacing serine with glycine has only a limited effect, probably because these residues have similar hydrophobicity. In contrast, Uy192 and D2 also differed at two positions (G11S and L13F) but the engineered version was more potent and the hemolytic activity was lower, even though the same pairs of residues were exchanged (Table 3), suggesting that the size of the side chains may have affected the conformation of the peptide in a favorable manner [19].

Although these peptides showed promising antibacterial activity, they were inactive towards phytopathogenic fungi. Similarly, opisin from *Opistophthalmus glabrifrons* was able to inhibit a range of bacteria such as *S. aureus*, *M. luteus*, *Bacillus megaterium, B. thuringiensis* and *Nocardia coralline*, but showed only a weak activity against *Candida tropicalis* [33]. One possible reason for such specificity towards bacteria is the effective electrostatic interaction of scorpion AMPs with the bacterial membrane—as a determinant factor for the bacterial viability—whereas acting against fungi via targeting cell wall components and/or intracellular molecules needs more complex interactions, which might be achieved through the synergism between different peptides [34,35]. 

In summary, our rational engineering approach yielded a number of derivative peptides with therapeutic indices better than the natural parental peptides. In particular, peptides D4, D5, D10 and D11 showed more potent antimicrobial activity and lower hemolytic and cytotoxic activity. D10 and D11 showed the highest activity against diverse bacteria and would be suitable as leads for the development of a new generation of antimicrobial products to address the prevalent threat of multidrug-resistant bacterial pathogens. 

## 4. Materials and Methods

### 4.1. Natural and Engineered AMPs

DNA sequences corresponding to the UyCT peptides were isolated from a cDNA library constructed from *U. yaschenkoi* telson total RNA [18]. The Uy17, Uy192 and Uy234 sequences were found in the transcriptome of the *U. yaschenkoi* venom gland [17]. The *U. manicatus* Um1, Um2, Um3, Um4 and Um5 sequences were previously described [20]. Engineered AMPs (D1–D12) were prepared based on the inherent structural characteristics of the sequences of UyCTx, Uy192 and Uy234. After selecting the conserved residues, four major structural determinants were considered: hydrophobicity, net charge, amphipathicity and helical conformation. All of the engineered AMPs were C-terminally amidated. The amino acid sequences, molecular masses and structural parameters for the engineered and natural AMPs described herein are summarized in Table 1. We investigated 16 peptides in this study, and they were chemically synthetized by CASLO (Kongens Lyngby, Denmark).

### 4.2. Antimicrobial Activity

The minimal inhibitory concentration (MIC) of each AMPs was determined against the Gram-positive bacteria *Listeria monocytogenes* (DSM20600), *L. grayi* (DSM20601), *L. fleischmannii* (DSM24998), *Staphylococcus aureus* (DSM2569) and *Micrococcus luteus* (DSM20030), and the Gram-negative species *Escherichia coli* (D31) and *Pseudomonas aeruginosa* (DSM50071). The scorpion peptides were prepared at concentrations of 0.015–250 μM. The assays were carried out in 384-well plates (Greiner Bio One, Frickenhausen, Germany) using Brain Heart Infusion Broth (BHIB) medium for *Listeria* spp., Tryptic Soy Broth (TSB) for *S. aureus*, and lysogeny broth (LB) for the others. The bacteria were cultivated overnight at either 37 °C (*Listeria* spp., *S. aureus*, *E. coli* and *P. aeruginosa*) or 30 °C (*M. luteus*) and subcultured in fresh media before the growth inhibition assays. Growth inhibition was measured as previously described [8]. Each assay was carried out at least three times and the MIC was recorded as the AMP concentration at which bacterial growth was totally inhibited (Figure S1).

### 4.3. Antifungal Assays

Antifungal activity was tested using *Fusarium culmorum* and *F. graminearum* strain 8/1 [34]. The fungi were cultured in the dark on Nirenberg Synthetic Nutrient Agar plates at 18 °C for 1–2 weeks and the harvested spores were incubated in tap water supplemented with 2–200 µM of each AMP at room temperature for 24 h in the dark [8,35]. The antifungal MIC was recorded as the AMP concentration at which spore germination was totally inhibited.

### 4.4. Hemolytic Activity

The hemolytic activity of each AMP was determined using pig erythrocytes [19]. Serial dilutions (6.25–100 µM) of each AMP were prepared, and after incubation for 1 h at 37 °C the cells were centrifuged and the absorbance of the supernatant was measured at 570 nm using an Eon Microplate Spectrophotometer (BioTek Instruments, Winooski, VT, USA). Three independent experiments were carried out for each concentration in triplicate. PBS and 10% Triton X–100 were used as negative and positive controls, respectively. The percentage hemolysis was calculated using the formula 100((A_peptide_–A_PBS_)/(A_triton_–A_PBS_)). The AMP concentration causing 50% hemolysis (HC_50_) of the pig erythrocytes was determined by non-linear regression with the data fitted to the logistical sigmoidal equation, using GraphPad Prism v5.0 (GraphPad Software, San Diego, CA, USA) (Figure S2).

### 4.5. Cytotoxic Activity

Cytotoxicity was evaluated using the baby hamster kidney cell line (BHK-21) included in F2H^®^-Kit Basic (Chromotek, Planegg-Martinsried, Germany). The BHK-21 cells were grown in 25-cm^2^ cell culture flasks (Greiner Bio One, Frickenhausen, Germany) containing Dulbecco’s Modified Eagle’s Medium (DMEM) supplemented with 4.5 g/L glucose, 110 mg/L sodium pyruvate and L-glutamine, and 10% fetal bovine serum (FBS), and were maintained in an NU-5810 incubator (ibs tecnomara, Fernwald, Germany) at 37 °C with a 5% CO_2_ atmosphere. The cells were subcultured at ~90% confluence by detaching with 0.25% trypsin and 0.03% EDTA (Sigma-Aldrich, St Louis, MO, USA). The AMPs were dissolved in water and diluted to final concentrations of 0.1, 1, 10 and 100 µM in DMEM.

BHK-21 cells were prepared and tested as previously described [36,37]. Confluent cells were detached, counted and seeded at a concentration of 8.5 × 10^4^ cell/mL in a 96-well culture plate (Greiner Bio One-Cell Star) 48 h before each experiment. The cells were rinsed with 100 µL PBS before adding 100 µL of the AMP solution and incubating for 2 h at 37 °C. The AMP solution was then removed and the cells were rinsed again with PBS before staining with 10% v/v alamarBlue™ Dye (Thermo Fisher Scientific, Schwerte, Germany) in DMEM for 1 h. The fluorescence of the metabolized alamarBlue™ Dye was measured in an Eon microplate reader using excitation and emission filters of 528 and 590 nm, respectively. DMEM and cells exposed to DMEM without AMPs were included as a blank reference and negative control, respectively. Four independent experiments were carried out for each AMP concentration in triplicate. To assess the percentage of viable cells compared to the control, the data were analyzed with GraphPad Prism v5.0 software using non-linear regression to fit the curve (Figure S2). The EC_50_ was reported as the AMP concentration that reduces cell viability by 50%.

### 4.6. Phylogenetic Analysis

The amino acid sequences were aligned using Clustal Omega [38]. To assess the evolutionary distance between scorpion AMPs, a phylogenetic tree was built using ClustalW2 Phylogeny on the EMBL-EBI platform with the Nexus UPGMA model. All natural scorpion AMPs with observed activities were included in the tree.

## Figures and Tables

**Table 1:** Native and engineered scorpion AMPs used in this study. All AMPs were C-terminally amidated.

AMP	Amino Acid Sequence	MW (Da)	Length	Net Charge	GRAVY^+^	Accession No.
UyCT1	GFWGKLWEGVKNAI	1603.9	14	+2	−0.050	AGA82754
UyCT3	ILSAIWSGIKSLF	1433.7	13	+2	1.392	AGA82755
UyCT5	IWSAIWSGIKGLL	1442.7	13	+2	1.138	AGA82756
Uy17	ILSAIWSGIKGLL	1369.43	13	+2	1.500	SRP045734 *
Uy192	FLSTIWNGIKGLL	1459.98	13	+2	0.969	SRP045734 *
Uy234	FPFLLSLIPSAISAIKRL	1986.19	18	+3	1.328	SRP045734 *
Um2	ISQSDAILSAIWSGIKSLF	2034.56	19	+1	0.832	JAA98072
Um3	GFWGKLWEGVKSAI	1577.23	14	+2	0.143	JAA98071
Um4	FFSALLSGIKSLF	1428.58	13	+2	1.492	JAA98071
Um5	IFKAIWSGIKSLF	1508.82	13	+3	1.077	JAA98069
D1	IFGAIWSGIKSLF	1437.11	13	+2	1.346	–
D2	FLSTIWNGIKSLF	1524.00	13	+2	0.862	–
D4	GFWGKLWKPVKKAI	1657.87	14	+5	−0.193	–
D5	GFWGKLLEGVKKAI	1544.52	14	+3	0.257	–
D10	FPFLKLSLKIPKSAIKSAIKRL	2497.71	22	+7	0.377	–
D11	GFWGKLWEGVKNAIKKK	1987.55	17	+5	−0.729	–

* High-throughput DNA and RNA sequence read archive (SRA); the peptides were taken from the transcriptome of *Urodacus yaschenkoi* [17].

**Table 2:** The engineered AMPs along with their parent sequences used in this study. The changed residues are shown in bold.

Peptide	Sequence
UyCT3	ILSAIWSGIKSLF
D1	I**FG**AIWSGIKSLF
Uy192	FLSTIWNGIKGLL
D2	FLSTIWNGIK**S**L**F**
UyCT1	GFWGKLWEGVKNAI
D4	GFWGKLW**KP**VK**K**AI
D5	GFWGKL**L**EGVK**K**AI
D11	GFWGKLWEGVKNAI**KKK**
D12	GFW**K**GKLW**K**EGVKNAI**K**
Uy234	FPFL-LSL-IP-SAI-SAIKRL
D10	FPFL**K**LSL**K**IP**K**SAI**K**SAIKRL

**Table 3:** The antimicrobial, cytotoxic and hemolytic profiles of native and engineered scorpion AMPs. MIC values were determined against seven different bacterial strains, cytotoxic values (ECx) against baby hamster kidney cells (BHK21) cells and hemolytic activity (HCx) towards pig erythrocytes. The values are the concentrations in µM. Values are the average of at least three independent experiments (*n* ≥ 3).

AMP	*M. luteus*	*E. coli*	*S. aureus*	*L. grayi*	*L. fleischmannii*	*L. monocytogenes*	EC_10_	EC_50_	EC_90_	HC_10_	HC_50_	HC_90_
**UyCT1**	1	4	–	4	4	4	0.65	17.37	52.12	31.5	142.50	644.46
**UyCT3**	4	8	–	8	4	4	0.57	15.37	46.12	15.78	58.15	214.22
**UyCT5**	4	15	8	8	8	4	0.91	24.55	73.65	2.39	20.59	177.49
**Uy17**	15	–	30	–	–	15	2.73	73.63	220.80	26.65	138.40	718.55
**Uy192**	15	15	15	15	4	8	1.92	51.95	155.90	35.85	155.6	675.22
**Uy234**	2	–	–	4	–	2	0.23	6.29	18.86	55.14	104.50	198.04
**Um2**	4	–	–	–	–	–	0.58	15.71	47.12	2.36	129	7033.22
**Um3**	2	15	15	4	4	8	2.359	63.68	191.1	70.48	126.2	246.84
**Um4**	8	8	15	15	4	8	3.22	87.01	261	n.c.	n.c. (100)	n.c.
**Um5**	2	15	–	2	2	2	0.36	9.71	29.12	11.944	59.25	293.90
**D1**	4	8	–	8	4	4	0.69	18.58	55.75	15.50	48	148.51
**D2**	4	–	8	–	15	15	13.95	376.70	1130	2.94	24.48	200.83
**D4**	2	–	–	8	15	30	36.30	980	2940	n.c.	n.c. (100)	n.c.
**D5**	8	15	–	8	15	15	2.50	67.63	202.90	110.86	1636	24,141.72
**D10**	0.25	8	–	8	30	30	5.24	141.40	424.30	149.75	1726	19,894.17
**D11**	0.25	4	–	1	1	2	0.66	17.81	53.43	39.76	1110	30,984.53

EC: effective cytotoxic concentration; HC: hemolytic concentration; n.c.: not convergent; n.c. (100): not convergent at 100 µM.

**Table 4:** Therapeutic indices (TI) of the native and engineered scorpion AMPs. The indices were calculated based on HC_50_ values.

AMP	*M. luteus*	*E. coli*	*S. aureus*	*L. grayi*	*L. fleischmannii*	*L. monocytogenes*
**UyCT1**	142.50	35.62	-	35.62	35.62	35.62
**UyCT3**	14.53	7.26	-	7.26	14.53	14.53
**UyCT5**	5.14	1.37	2.57	2.57	2.57	5.14
**Uy17**	9.22	-	4.61	-	-	9.22
**Uy192**	10.37	10.37	10.37	10.37	38.90	19.45
**Uy234**	52.25	-	-	26.12	-	52.25
**Um2**	32.25	-	-	-	-	-
**Um3**	63.10	8.41	8.41	31.55	31.55	15.77
**Um4**	-	-	-	-	-	-
**Um5**	29.62	3.95	-	29.62	29.62	29.62
**D1**	11.99	5.99	-	5.99	11.99	11.99
**D2**	6.12	-	3.06	-	1.63	1.63
**D4**	-	-	-	-	-	-
**D5**	204.50	109.06	-	204.50	109.06	109.06
**D10**	6904	215.75	-	215.75	57.53	57.53
**D11**	4440	277.5	-	1110	1110	555

## Supplementary Materials

The following are available online at www.mdpi.com/2072-6651/9/1/22/s1, Figure S1: Growth inhibition assays, Figure S2: Cytotoxicity and Hemolysis graphs.
