# Supplementary material for: Bioactivity of Natural and Engineered Antimicrobial Peptides from Venom of the Scorpions Urodacus yaschenkoi and U. manicatus
Source: Toxins (Basel). 2017 Jan 6;9(1):22. doi: 10.3390/toxins9010022 (PMC5308254; doi:10.3390/toxins9010022)
Supplement: Supplementary file 1 [file toxins-09-00022-s001.pdf]

Supplementary Materials: Bioactivity of Natural and Engineered Antimicrobial Peptides from Venom of the Scorpions *Urodacus yaschenkoi* and *U. manicatus*

Karen Luna-Ramirez, Miray Tonk, Mohammad Rahnamaeian and Andreas Vilcinskas

The MIC values are presented based on results of the growth curve assays. In contrast to the inhibition zone-based assays that are carried out on disks, such values are usually reported in tables as definite numbers without including the standard errors [1–4]. However, to support our presented data on MIC values, we provide two graphs (Figure S1 for MIC) as the representatives of all combinations (96 combinations including all natural and engineered peptides: 16 peptides against 6 bacteria) we tested to avoid unnecessary presentation of many comparable graphs.

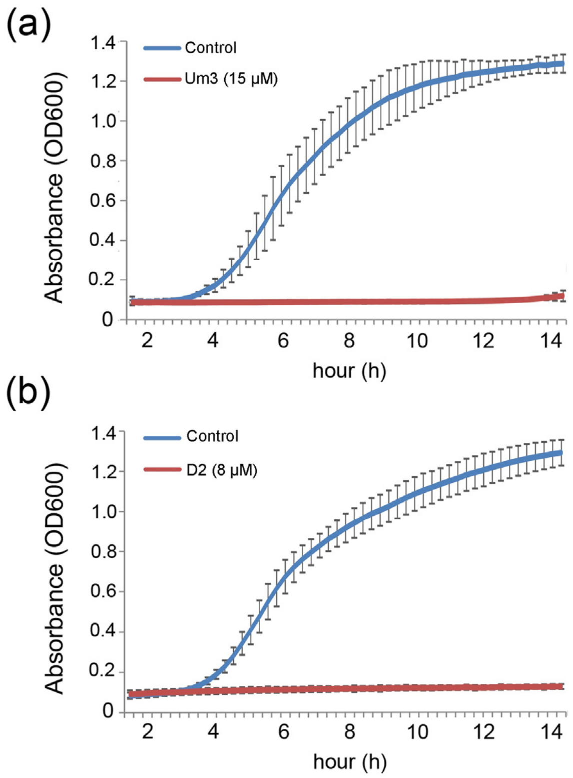

Figure S1. Growth inhibition assays. (**a**) *Escherichia coli* strain D31 in mid-logarithmic phase was incubated with medium (control) or with natural peptide Um3 (15 µM). (**b**) *Staphylococcus aureus* in mid-logarithmic phase was incubated with medium (control) or with engineered peptides D2 (8 µM). The growth rate was determined by measuring the optical density (OD) of the culture at 600 nm. Values are the mean ± SD. The experiments were carried out at least three times.

Cytotoxicity and Hemolysis

Data of at least three different experiments with its replicates was average and error (SEM) was calculated (raw data graph). Then, data was fitted with a non-linear fit using GraphPad Prism. Figure S2 shows graphs for cytotoxicity and hemolysis determinations. Raw data for both cytotoxicity and hemolysis are presented for all peptides tested herein (left panel); while for the fitted curves, only four peptides are shown as representatives off all peptides used in this study. This was done in order to present clearer and easy to read graphs.

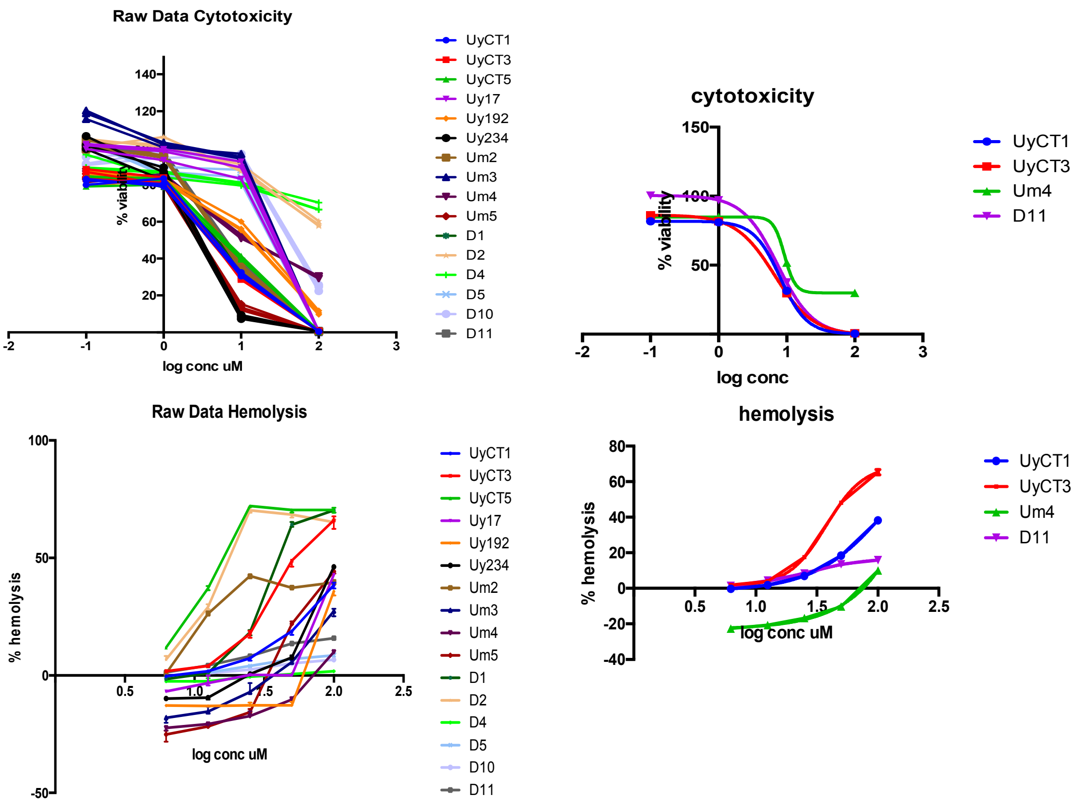

Figure S2. Cytotoxicity and Hemolysis graphs. Raw data for all peptides used in this study (left panel) and representatives of fitted curves (right panel).

References

1. Hu, F.; Wu, Q.; Song, S.; She, R.; Zhao, Y.; Yang, Y.; Zhang, M.; Du, F.; Soomro, M.H.; Shi, R. Antimicrobial activity and safety evaluation of peptides isolated from the hemoglobin of chickens. *BMC Microbiol.* **2016**, *16*, 287.
2. Pedron, C.N.; Torres, M.T.; Lima, J.A.; Silva, P.I.; Silva, F.D.; Oliveira, V.X. Novel designed VmCT1 analogs with increased antimicrobial activity. *Eur. J. Med. Chem.* **2016**, *126*, 456–463.
3. Castellano, P.; Mora, L.; Escudero, E.; Vignolo, G.; Aznar, R.; Toldrá, F. Antilisterial peptides from Spanish dry-cured hams: Purification and identification. *Food Microbiol.* **2016**, *59*, 133–141.
4. Bolouri Moghaddam, M.R.; Tonk, M.; Schreiber, C.; Salzig, D.; Czermak, P.; Vilcinskas, A.; Rahnamaeian, M. The potential of the Galleria mellonella innate immune system is maximized by the co-presentation of diverse antimicrobial peptides. *Biol. Chem.* **2016**, *397*, 939–945.
